# Canagliflozin mitigates ferroptosis and improves myocardial oxidative stress in mice with diabetic cardiomyopathy

**DOI:** 10.3389/fendo.2022.1011669

**Published:** 2022-10-14

**Authors:** Shuqin Du, Hanqiang Shi, Lie Xiong, Ping Wang, Yanbo Shi

**Affiliations:** ^1^ Central Laboratory of Molecular Medicine Research Center, Jiaxing Traditional Chinese Medicine (TCM) Hospital Affiliated to Zhejiang University of Traditional Chinese Medicine, Jiaxing, China; ^2^ Jiaxing Key Laboratory of Diabetic Angiopathy Research, Jiaxing, China; ^3^ School of Pharmacy, Zhejiang University of Technology, Hangzhou, China; ^4^ School of Medicine, Jiaxing University, Jiaxing, China

**Keywords:** canagliflozin, sodium-glucose cotransporter 2 inhibitor, ferroptosis, mitochondria, diabetic cardiomyopathy, lipid peroxidation

## Abstract

Canagliflozin (Cana), an anti-diabetes drug belongs to sodium-glucose cotransporter 2 inhibitor, is gaining interest because of its extra cardiovascular benefits. Ferroptosis is a new mode of cell death, which can promote the occurrence of diabetic cardiomyopathy (DCM). Whether Cana can alleviate DCM by inhibiting ferroptosis is the focus of this study. Here, we induced DCM models in diabetic C57BL6 mice and treated with Cana. Meanwhile, in order to exclude its hypoglycemic effect, the high glucose model in H9C2 cells were established. In the *in vivo* study, we observed that Cana could effectively alleviate the damage of cardiac function in DCM mice, including the increasing of lactate dehydrogenase (LDH) and cardiac troponin I (cTnI), the alleviating of myocardial fiber breakage, inflammation, collagen fiber deposition and mitochondrial structural disorder. We evaluated reactive oxygen species (ROS) levels by DCFH-DA and BODIPY 581/591 C11, *in vitro* Cana reduced ROS and lipid ROS in H9C2 cells induced by high glucose. Meanwhile, JC-1 fluorochrome assay showed that the decreased mitochondrial membrane potential (MMP) was increased by Cana. Furthermore, the inhibitory effects of Cana on myocardial oxidative stress and ferroptosis were verified *in vivo* and *in vitro* by protein carbonyl (PCO), malondialdehyde (MDA), superoxide dismutase (SOD), catalase (CAT), glutathione (GSH). As a key inducer of ferroptosis, the deposition of total iron and Fe^2+^ can be inhibited by Cana both *in vivo* and *in vitro*. In addition, western blot results indicated that the expression of ferritin heavy-chain (FTN-H) was down-regulated, and cystine-glutamate antiporter (xCT) was up-regulated by Cana in DCM mice and cells, suggesting that Cana inhibit ferroptosis by balancing cardiac iron homeostasis and promoting the system Xc^-^/GSH/GPX4 axis in DCM. These findings underscore the fact that ferroptosis plays an important role in the development and progression of DCM and targeting ferroptosis may be a novel strategy for prevention and treatment. In conclusion, Cana may exert some of its cardiovascular benefits by attenuating ferroptosis.

## Graphical Abstract

Canagliflozin mitigates ferroptosis and improves myocardial oxidative stress in mice with diabetic cardiomyopathy.
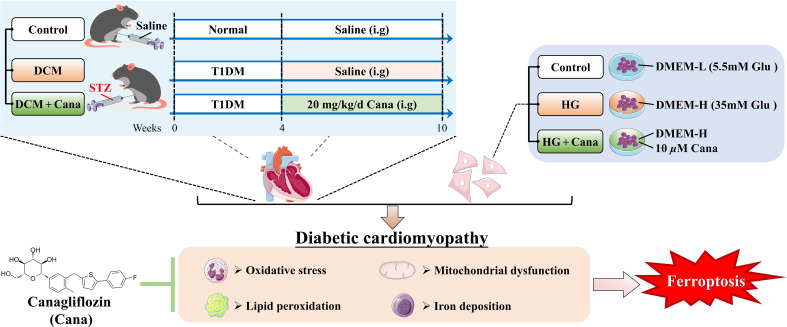

## Introduction

Diabetes is a metabolic disease characterized by chronic hyperglycemia. Long-term hyperglycemia will aggravate the damage of the patient’s systemic tissues and organs, leading to a variety of serious complications. It is reported that the probability of cardiovascular disease in diabetic patients is 2-3 times higher than that in non-diabetic patients (1), and diabetic cardiovascular disease has become an important factor causing death and disability in diabetic patients. Among them, diabetic cardiomyopathy (DCM) is a diabetic based disease that occurs in the absence of other cardiac dysfunction and eventually leads to heart failure. However, the pathogenesis of DCM has not been fully elucidated. Because of the complexity and harmfulness, the prevention and treatment of the disease is imminent.

As an essential trace element in almost all living organisms, iron plays a critical role in maintaining normal physiological function. During the past decades, more and more attention has been paid to the iron overload related diseases. In diabetes, persistent high blood glucose and insulin resistance can cause a vicious circle by altering cellular metabolism, promoting the accumulation of peroxidation and the death of cells. So far, diabetes has been verified to be associated with abnormal iron metabolism. For example, systemic iron overload can contribute to abnormal glucose metabolism and the onset of type 2 diabetes (T2DM) (2) and aggravate insulin resistance (3). Furthermore, it is confirmed that ferroptosis, a programmed cell death which characterized by iron-dependent accumulation of lethal lipid peroxidation (4), is involved in cognitive impairment and diabetic endothelial dysfunction in type I diabetes mellitus (T1DM) (5, 6). In particularly, a growing number of evidence supports that maintenance of iron homeostasis is essential for proper cardiac function since the iron accumulation will induce ferroptosis and leading to the damage and dysfunction of myocardial tissues (7). Recently, Cai group (8) identified the role of ferroptosis in DCM, and reported that Nrf2 activation by sulforaphane inhibited ferroptosis and prevented DCM, suggesting that it is feasible to treat DCM by inhibiting ferroptosis. Due to the limited regenerative capacity of the myocardium in mammalian adult hearts, inhibition of cardiomyocyte death might be one of the important ways to alleviate DCM (9). Taken together, taking ferroptosis as the starting point may provide a new strategy for the prevention and control of DCM.

Canagliflozin (Cana), a hypoglycemic drug of sodium-glucose cotransporter 2 inhibitor (SGLT2i), has shown promising anti-cardiovascular effect in multicenter clinical randomized double-blind studies, which can reduce the risk of cardiovascular death or hospitalization for heart failure in diabetic patients and non-diabetic patients (10, 11). Meanwhile, Cana have shown the ability to attenuate oxidative stress and improve myocardial function by suppressing apoptosis, promoting antioxidant and anti-inflammatory pathways (11, 12). At present, the protective effects of Cana on the heart have been gradually revealed in various myocardial injuries, such as autoimmune myocarditis (13), myocardial lipotoxicity (12) and even isoprenaline-induced cardiotoxicity (14). In addition, a recent study reported that Cana may exert its cardiovascular benefits partly *via* its mitigation of ferroptosis (15), which is similar to the effect of empagliflozin (16), another SGLT2i. These studies provide clues that regulating ferroptosis may play an important role in the cardioprotective effects of SGLT2is. However, the cardioprotective mechanisms of Cana against DCM remain unclear and still need to be further explored.

Here, we aimed to determine whether the SGLT2i Cana plays a role in protection against ferroptosis in DCM *in vivo* and *in vitro*. We addressed this question using our established *in vivo* model of DCM induced by diabetic mice and high glucose induced H9C2 cells injury model *in vitro* to explore the potential mechanism of Cana in the treatment of DCM.

## Method

### Animals’ treatment

Male C57BL/6J mice aged 6-8 weeks with weights of 18-20 g were obtained from the Slack Laboratory Animal Co., Ltd. (Shanghai, China). Production license number: SCXK (Shanghai) 2017-0005. The mice were kept in the Experimental Animal Center of Zhejiang University of Technology under specific-pathogen-free (SPF) environment (room temperature 22 ± 2°C, humidity 55 ± 5%, 12 h light/12 h dark cycle), with unrestricted access to food and water. Experiments were carried out according to the Guideline for the Care and Use of Laboratory Animals published by the National Institute of Health, USA. All experimental procedures were approved by the Animal Experimental Ethics Committee of Zhejiang University of Technology (Animal experimental research plan No. 20220317021).

Mice were allowed to acclimatize in the laboratory environment for 1 week before the beginning of the experiment. DCM model establishment: The mice were given a single intraperitoneal injection of 150 mg/kg 1% streptozotocin (STZ, V900890, Sigma, USA, dissolved in 0.1 mol/L sodium citrate buffer, pH = 4.4 - 4.6) (17, 18). Mouse blood from the tail vein was collected in each group of the model mice and tested by glucose meter (Accu-Chek^®^ Performa test strips, Roche, Accu-Chek^®^ Performa Combo, Roche, USA) on day 3, 5 and 7 after injection. The mice with random blood glucose levels ≥ 16.7 mmol/L were considered as diabetic models, and then kept for 4 weeks to induce DCM. Randomization was used to divide the mice into three groups (n = 8 per group): (1) CON group: mice were administered intragastrically with saline only. (2) DCM group: DCM model mice were treated with saline for 6 weeks. (3) DCM + Cana group: DCM model mice were treated with 20 mg/kg/d canagliflozin (15, 19, 20) (C126191, Aladdin, China) for 6 weeks. On the last day, electrocardiogram examination was performed after the drug intervention, then all animals were weighted and sacrificed, heart tissues were removed freshly and aseptically for measurements.

### Cells treatment

H9C2 rat cardiomyocytes were obtained from the National Infrastructure of Cell Line Resource (1101RAT-PUMc000219). H9C2 were cultured in DMEM medium with 5.5 mmol/L glucose (11885084, Gibco, USA), 10% fetal bovine serum (FBS, 10100147, Gibco, USA) and 100 U/mL penicillin-100 *μ*g/mL streptomycin sulfate (B540732, Sangon, China) at 37° C under 5% CO_2_. According to the literature (4, 21, 22), in this research, high glucose was defined as 35 mmol/L was used to intervene H9C2 cardiomyocytes for 24 h to establish a DCM model *in vitro* study.

H9C2 cells were divided into three groups: (1) CON group: H9C2 cells were cultured with DMEM medium containing 5.5 mmol/L glucose. (2) HG group: H9C2 cells were cultured with DMEM medium containing 35 mmol/L glucose. (3) HG + Cana group: H9C2 cells were cultured with DMEM medium containing 35 mmol/L glucose and a final concentration of 10 *μ*M canagliflozin. After 24 h of treatment, the H9C2 cells in each group were measured for related indexes.

### Electrocardiogram

The mice were anesthetized using 3% sodium pentobarbital (50 mg/kg), and then fixed on a table in the supine position. Subcutaneous needle electrodes were connected to the mice for the limb lead at position II and electrocardiograms were recorded using the Power Lab 26 T data acquisition systems (AD, China). The ECG parameters PP interval, PR interval, RR interval, QRS interval and heart rate were recorded.

### Collection of blood and tissue samples

At the end of the medical intervention, the mice were anesthetized using 3% sodium pentobarbital (50 mg/kg). Blood was collected through pericardiocentesis and then centrifuged at 4500 g (4°C) for 15 min to separate serum. Serum was stored at -80°C until analysis. Cardiac tissues were collected, the left ventricle tissues of the hearts were fixed in a 4% paraformaldehyde (E672002, Sangon, China) or 2.5% glutaraldehyde (A17876, Alfa Aesar, USA) for subsequent histopathology analysis and transmission electron microscopy (TEM). The remaining tissue samples were rapidly frozen with liquid nitrogen and stored at -80°C for futher examination.

### Histopathology

The left ventricle tissues of the hearts were fixed in 4% paraformaldehyde (E672002, Sangon, China) for 48 h and embedded in paraffin, 5 *μ*m sections were cut longitudinally and stained with hematoxylin and eosin (HE) to evaluate the pathological changes of myocardial tissue. Masson trichrome staining and Sirus red staining were used to observe the deposition of collagen in myocardial tissue. Image J software (https://imagej.nih.gov/ij/) was used for quantitatively analysis the degree of myocardial fibrosis.

### Transmission electron microscopy 

The left ventricle tissue was fixed in 2.5% glutaraldehyde (A17876, Alfa Aesar, USA). After fixation, dehydration, and impregnation, samples were embedded into epoxy resins and acrylic resins, 1 *μ*m-thick sections were cut on an ultramicrotome with glass knives, then myofilament and mitochondria were observed by transmission electron microscope (Hitachi H-7500, Japan).

### Cell viability assay

Cell viability was assessed using Cell Counting Kit-8 (CCK-8 kit, E606335, Sangon, China). Briefly, H9C2 cells were seeded into 96-well plates at 2×10^3^/well. After overnight adhesion, H9C2 cells were starved for 6 h by serum-free medium to sync the cell cycle, and then cells were exposed to different concentrations (0.5, 2.5, 5, 10, 20, 40 *μ*M) of Cana for 24 h. Subsequently, 10 *μ*L CCK-8 solution was added to each well and incubated for 2 h at 37°C. The absorbance at 450nm was measured by a multiskan spectrum (Multiskan GO, Thermo, USA).

### Biochemical analysis

All kits used in the determination of cTnI, MDA, PCO, GSH, ATP, Fe^2+^, total iron and glucose contents are listed as below: mouse cardiac troponin I (cTnI) assay kit (ML989022-J, Enzyme-Linked Biotechnology, China), malondialdehyde (MDA) content assay kit (BC0025, Solarbio, China), protein carbonylation (PCO) assay kit (ML076345, Enzyme-Linked Biotechnology, China), reduced glutathione (GSH) content assay kit (D799614, Sangon, China), ATP assay kit (S0026, Beyotime, China), ferrous ion colorimetric assay kit (E-BC-K304-S, Elabsciences, China), total iron colorimetric assay kit (E-BC-K772-M, Elabsciences, China) and glucose assay kit with O-toluidine (S0201M, Beyotime, China). All experiments were performed according to the manufacturers’ instructions, and measured by multiskan spectrum (Multiskan GO, Thermo, USA), luminometer (GloMax 20/20, Promega, USA) or spectrophotometer (Quick Drop, Molecular Devices, USA), respectively.

### Detection of enzyme activities

The methods of activities assay of LDH, CAT, SOD, GSH-Px were listed as following: lactate dehydrogenase (LDH) activity assay kit (D799208, Sangon, China), catalase (CAT) activity assay kit (D799598, Sangon, China), total superoxide dismutase (SOD) assay kit with WST-8 (S0101S, Beyotime, China) and total glutathione peroxidase (GSH-Px) assay kit with NADPH (S0058, Beyotime, China). All experiments were performed according to the manufacturers’ instructions, and measured by Multiskan Spectrum (Multiskan GO, Thermo, USA).

### Fluorescence staining assay

Superoxide production, lipid peroxidation and MMP were detected using 2’,7’-dichlorofluorescein diacetate (DCFH-DA, 35845, Sigma, USA), BODIPY 581/591 C11 (D3861, Thermo, USA) and JC-1 fluorochrome (C2006, Beyotime, China) respectively. H9C2 cells were seeded in 6-well plates at 10×10^4^/well. After the treatments as described in section of “Cells treatment”, H9C2 cells were washed twice with PBS, followed by incubation with 5 *µ*M DCFH-DA, 2 *µ*M BODIPY 581/591 C11 or 5 *µ*M JC-1 for 30 min at 37°C in darkness, then the cells were washed three times with PBS or JC-1 staining buffer, respectively. Finally, fluorescence was measured using inverted fluorescence microscope (Axio Observer D1, ZEISS, Germany), and the fluorescence intensity of each group was analyzed by using Image J software (https://imagej.nih.gov/ij/).

### Quantitative PCR

Total RNA was extracted from cardiac tissues or H9C2 cells by using Trizol lysate (9109, Takara, Japan), and subjected to reversely transcribed into cDNA using PrimeScript™ RT Master Mix (RR036Q, Takara, Japan) according to the manufacturers’ instructions. The qPCR assay was performed on Real-Time PCR system (7500, ABI, USA) with 95° C for 30 s, 1 cycle: 95° C for 5 s, 60° C for 34 s, 40 cycles (RR820, Takara, Japan). GAPDH was used as internal reference gene, the primer sequences were listed in **Table 1**. The relative gene expressions were determined using the 2^-ΔΔCt^ method.

**Table 1:** qPCR Primer sequences.

Gene	Forward sequence (5’- 3’)	Reverse sequence (5’- 3’)	ProductSize (bp)
Rat_TfR1	CGGCTACCTGGGCTATTGTA	TTCTGACTTGTCCGCCTCTT	84
Rat_FPN	TCCTGGGCTTCGACTGTATC	CAAGTGAAGGCCACAGTTCC	124
Rat_FTN-H	GGCTGAATGCAATGGAGTGT	TCTTGCGTAAGTTGGTCACG	186
Rat_SLC7A11	GGTGGTGTGTTTGCTGTCT	AGAGGAGTGTGCTTGTGGA	101
Rat_GPX4	AATTCGCAGCCAAGGACATC	GGCCAGGATTCGTAAACCAC	170
Rat_GAPDH	CAATCCTGGGCGGTACAACT	TACGGCCAAATCCGTTCACA	162
Mouse_TfR1	TTGGGTAGTTGGAGATTGCC	TGAGGTCTTTGGCTTCTGGT	247
Mouse_FPN	AAGCGGCCCACACTAAGAAA	AGGCAATGTCCCATGTTGGT	237
Mouse_FTN-H	CATCAACCGCCAGATCAACC	GTCATCACGGTCTGGTTTCTTTAT	220
Mouse_SLC7A11	GGCACCGTCATCGGATCAG	CTCCACAGGCAGACCAGAAAA	100
Mouse_GPX4	CGCGATGATTGGCGCT	CACACGAAAACCCCTGTACTTATCC	175
Mouse_GAPDH	GAAGGGCTCATGACCACAG	AGATCCACGACGGACACATT	221

TfR1, transferrin receptor 1; FPN, ferroportin; FTN-H, ferritin heavy-chain; SLC7A11, Solute carrier family 7 member 11; GPX4, glutathione peroxidase 4; GAPDH, glyceraldehyde-3-phosphate dehydrogenase.

### Western blotting

Western blotting analyses of cardiac tissue and H9C2 cells were performed as described. Briefly, total proteins of heart tissues or H9C2 cells were extracted with RIPA lysis buffer (R0020, Solarbio, China) containing protease inhibitor (04693116001, Roche, Germany). Then protein concentration was quantified by the BCA protein assay kit (C503051, Sangon, China). 30 *μ*g of total protein samples from different groups were first electrophoresed through 10% SDS-PAGE gels and then transferred onto the nitrocellulose membranes (F619511, Sangon, China). After blocked with 5% skim milk in TBST at room temperature for 1 h, membranes were washed and incubated with the following primary antibodies overnight (as shown in **Table 2**). Washed with TBST, followed by incubated with corresponding HRP-conjugated secondary antibodies at room temperature for 2 h. The target bands were subsequently detected with chemiluminescent (ECL) Kit (D601039, Sangon, China) and chemiluminescent imaging system (5200 multi, Tanon, China).

**Table 2:** The antibodies and dilution ratio.

Antibodies	Reacts with	Dilution ratio	Cat No	Manufacturers
anti-TfR1	Rat, Mouse	1:1000	ab84036	Abcam/UK
anti-FPN1	Rat, Mouse	1:500	ab58695	Abcam/UK
anti-FTN-H	Rat, Mouse	1:1000	ab75973	Abcam/UK
anti-GPX4	Rat, Mouse	1:1000	ab125066	Abcam/UK
anti-xCT	Rat, Mouse	1:1000	ab175186	Abcam/UK
anti-GAPDH	Rat, Mouse	1:10000	ab181602	Abcam/UK
HRP labeled Goat anti rabbit IgG (H + L)		1:50000	BK-R050	Bioker/China

TfR1, transferrin receptor 1; FPN, ferroportin; FTN-H, ferritin heavy-chain; xCT, cystine-glutamate antiporter; GPX4, glutathione peroxidase 4; GAPDH, glyceraldehyde-3-phosphate dehydrogenase.

### Statistical analysis

Measurement data were expressed as the mean ± standard deviation and analyzed by SPSS software (version 23.0, USA). Statistical comparisons were performed using one-way ANOVA. *P* < 0.05 was considered statistically significant.

## Result

### Cana attenuates myocardial injury in DCM mice

To investigate the therapeutic effect of Cana on myocardial injury in diabetes, mouse model of DCM was established. At the end of the experiment, the basic state and cardiac function of mice in these groups were faithfully recorded (**Table 3**). Mice in DCM group showed obvious diabetic symptoms such as elevated blood glucose, polydipsia, polyphagia, and weight loss. The results of ECG showed that significant slowing of heart rate accompanied by overt prolongation of RR interval, PP interval, and PR interval were present in DCM mice. At the same time, LDH and cTnI, both markers of cardiac injury, were also significantly increased in DCM mice, indicating that DCM indeed occurred. In contrast, the mice treated with Cana recovered both diabetic symptoms and cardiac function, although blood glucose levels remained higher than in controls. All these results indicated that Cana could alleviate diabetic myocardial injury in DCM mice.

**Table 3:** Basic characteristics of mice in each group at the end of experiment.

	CON	DCM	DCM + Cana
Blood glucose (mmol/L)	8.90 ± 0.47	31.28 ± 3.58^***^	18.26 ± 1.09^###^
Body weight (mg)	29.45 ± 2.26	17.34 ± 2.31^***^	20.11 ± 1.34^n.s.^
Food intake (g/24 h)	12.28 ± 0.34	24.83 ± 3.44^***^	15.12 ± 1.64^###^
Water intake (mL/24 h)	33.6 ± 1.62	116.82 ± 13.59^***^	81.50 ± 18.93^#^
RR interval (ms)	135 ± 8.94	171 ± 11.14^***^	155 ± 10.00^#^
HR (bpm)	446 ± 28.49	328 ± 39.38^***^	388 ± 22.86^#^
PP interval (ms)	122 ± 14.70	158 ± 6.32^***^	140 ± 7.48^#^
PR interval (ms)	36 ± 4.15	71 ± 19.08^***^	56 ± 4.90^#^
QRS interval (ms)	28 ± 2.45	32 ± 4.00^n.s.^	30 ± 3.16^n.s.^
LDH (U/mg tissue)	3.56 ± 1.01	21.32 ± 1.54^***^	1.91 ± 0.20^###^
cTnI (pg/mL)	11.65 ± 0.88	18.33 ± 1.86^**^	14.67 ± 1.04^#^

Data are expressed as mean ± standard deviation (n = 8). HR, heart rate; LDH, lactate dehydrogenase; cTnI, mouse cardiac troponin I. (*: P<0.05; **: P<0.01; ***: P<0.001; compared with CON group; #: P< 0.05; ##: P<0.01; ###: P<0.001; compared with DCM group; n.s.: no significance.)

### Cana inhibits diabetic myocardial tissues fibrosis and oxidative stress

**Figure 1:**
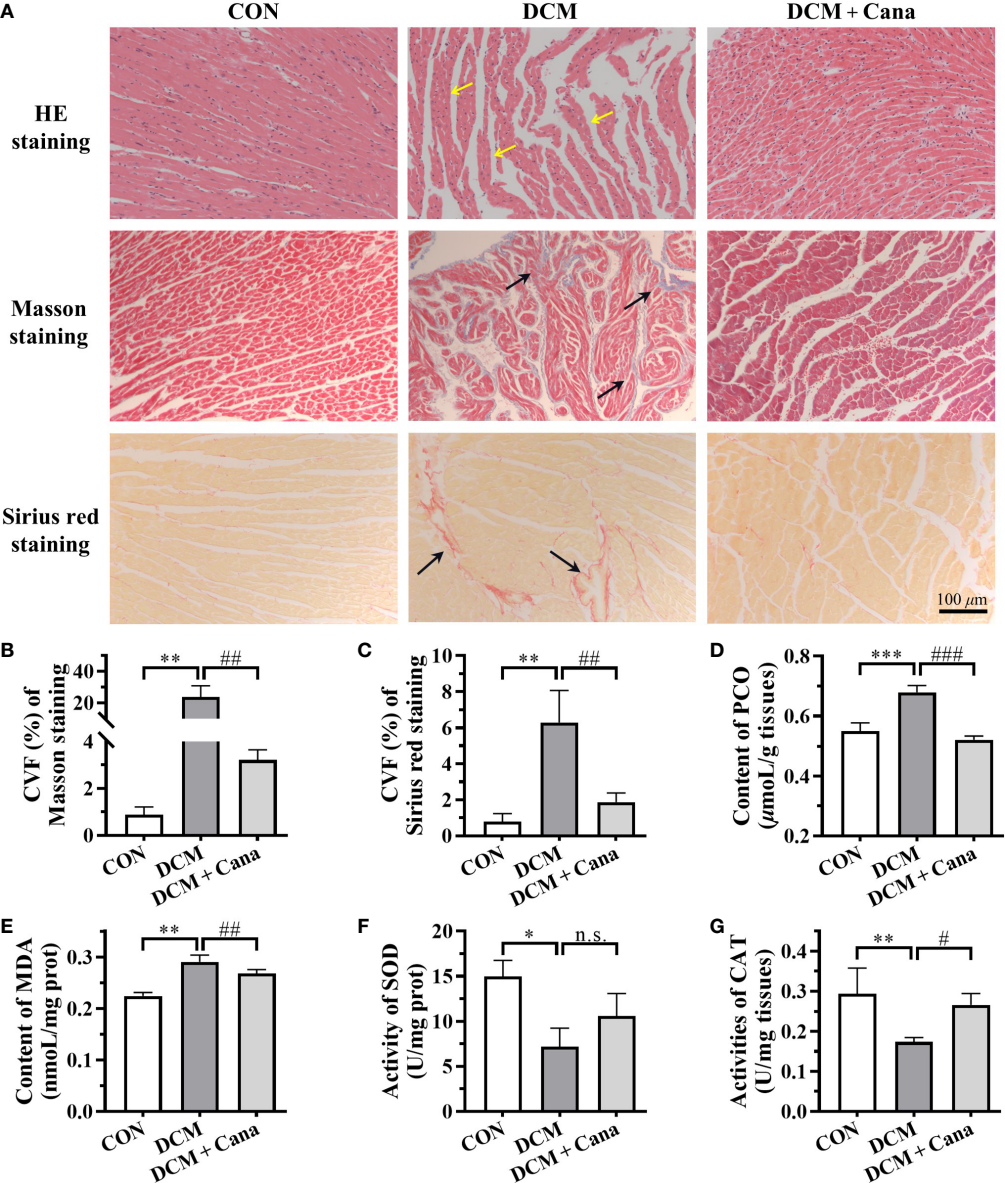
Canagliflozin inhibits oxidative stress and fibrosis in diabetic myocardial tissue **(A)** Pathological staining results of myocardial tissues (Scale bar: 100 *µ*m). **(B)** CVF of Masson staining. **(C)** CVF of Sirus red staining. Contents of **(D)** PCO and **(E)** MDA, activities of **(F)** SOD and **(G)** CAT in myocardial tissues. CVF, collagen volume fraction; PCO, protein carbonyl; MDA, malondialdehyde; SOD, superoxide dismutase; CAT, catalase. (*: *P* < 0.05; **: *P* < 0.01; ***: *P* < 0.001; compared with CON group; #: *P* < 0.05; ##: *P* < 0.01; ###: *P* < 0.001; compared with DCM group; n.s.: no significance.).

Pathological staining was used to further verify the degree of myocardial injury in mice. HE staining showed (**Figure 1A**) that myocardial fibers were intact and aligned in CON group, but disorganized or even fractured in the DCM group (yellow arrow). Besides, necrosis or inflammatory cells were increased, and the myocardial striations became blurred in DCM mice. In DCM + Cana group, myocardial fiber disorder was improved, and necrosis or inflammatory cells were obviously reduced. The increasing of collagen fibers in myocardial tissues were observed by Masson staining and Sirius red staining. As shown in **Figures 1A-C**, compared with CON group, many collagen fibers were deposited in myocardial tissues of DCM mice (black arrow), which was significantly alleviated after Cana treatment.

Next, we measured the level of oxidative stress, an inducer of DCM, in the myocardium of each group of mice. Compared with CON group, the contents of MDA and PCO in the myocardium of DCM mice were significantly increased (**Figures 1D, E**), while the activities of antioxidant enzymes SOD and CAT were obviously decreased (**Figures 1F, G**). And after treatment with Cana, the biochemical parameters of oxidative stress in DCM mice were improved, including the decrease of MDA and PCO (**Figures 1D, E**), and the increase of CAT (**Figure 1G**). These results suggested Cana exerted an anti-oxidative stress effect here.

### Cana mitigates mitochondrial damage and ferroptosis in DCM mice

**Figure 2:**
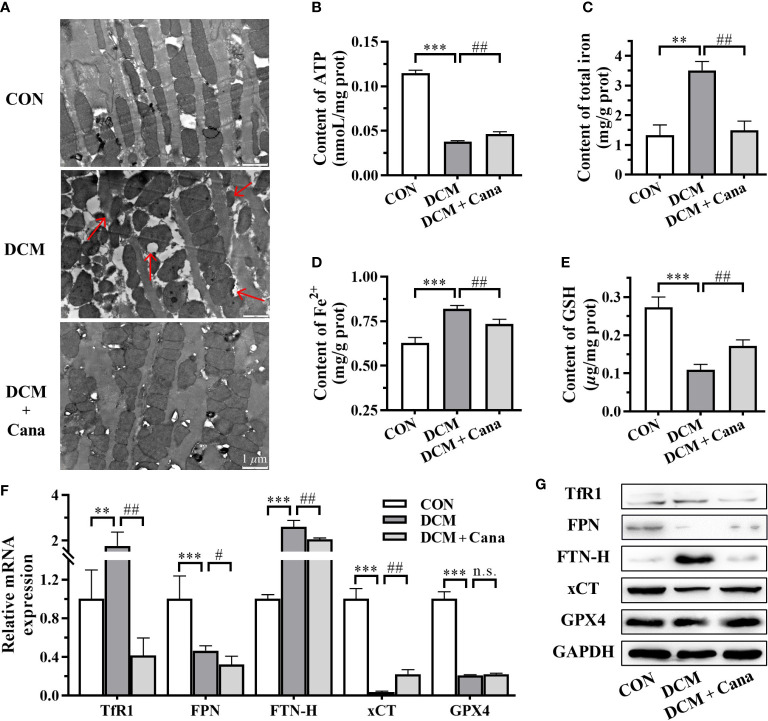
Canagliflozin inhibits mitochondrial damage and ferroptosis in diabetic mice. **(A)** Morphological and structural observation of mitochondria in myocardial tissues (Scale bar: 1 *µ*m). Contents of **(B)** ATP; **(C)** total iron; **(D)** Fe^2+^ and **(E)** GSH in myocardial tissues. **(F)** Transcriptional level and **(G)** protein level of iron metabolism and ferroptosis related genes examined by qPCR and western blotting assay. ATP, adenosine triphosphate; GSH, glutathione; TfR1: transferrin receptor 1; FPN, ferroportin; FTN-H, ferritin heavy-chain; xCT, cystine-glutamate antiporter; GPX4, glutathione peroxidase 4; GAPDH, glyceraldehyde-3-phosphate dehydrogenase. (**: P<0.01; ***: P<0.001; compared with CON group; #: P<0.05; ##: P<0.01; compared with DCM group; n.s.: no significance.)

TEM was applied to observe the changes of mitochondrial morphology and structure in myocardial tissues. The ultrastructure was obviously changed in myocardial tissues of DCM mice, the myofilaments were fragmented, the mitochondria were swollen, part of the outer membrane was ruptured, and the mitochondrial cristae were disappeared or broken (**Figure 2A**). However, the disturbance of mitochondrial structure in DCM + Cana group was effectively improved, the swollen mitochondria were less, the mitochondrial inner and outer membranes were intact, and the number of mitochondrial cristae increased (**Figure 2A**). Correspondingly, the insufficiency of ATP level in myocardial tissues of DCM mice was partially improved after Cana treatment (**Figure 2B**), which reflected that Cana could reduce mitochondrial damage and protect mitochondrial function to a certain extent.

Since the homeostasis metabolism of cardiac iron was closely related to heart disease, we examined key factors associated with iron metabolism and ferroptosis. In DCM mice, we found that the contents of total iron (**Figure 2C**) and Fe^2+^ (**Figure 2D**), and the expression of transferrin receptor 1 (TfR1) and ferritin heavy-chain (FTN-H) (**Figures 2F, G**) were significantly increased. Meanwhile, the content of GSH (**Figure 2E**) and the expression of ferroportin (FPN) and cystine-glutamate antiporter (xCT) (**Figures 2F, G**) were significantly reduced in DCM mice compared to normal mice. After the treatment of Cana, the contents of total iron (**Figure 2C**) and Fe^2+^ (**Figure 2D**), and the expression of TfR1 and FTN-H (**Figures 2F, G**) were all obviously lower, while the content of GSH (**Figure 2E**) and the expression of xCT (**Figures 2F, G**) were both significantly increased in DCM mice. But the protein level of glutathione peroxidase 4 (GPX4) showed no difference in the myocardial tissues between DCM group and DCM + Cana group (**Figure 2G**). These assays testified that ferroptosis indeed exist in DCM mice and Cana could inhibit ferroptosis by balancing iron homeostasis and increasing xCT expression.

### Cana alleviates high glucose injury of cardiomyocytes without hypoglycemic effect

**Figure 3:**
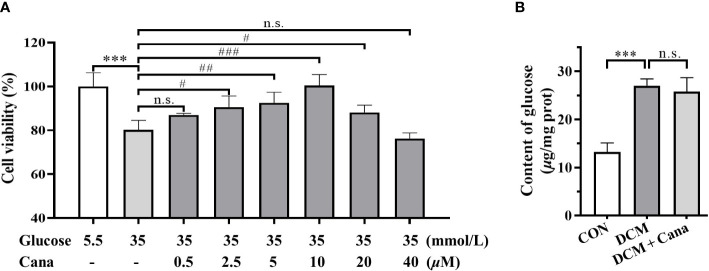
Canagliflozin alleviates high glucose injury of cardiomyocytes by non-hypoglycemic way. **(A)** Effects of high concentration of glucose and different concentrations of canagliflozin on the cell viability of H9C2 cells. **(B)** Content of glucose in H9C2 cells of each group. (***: P<0.001; compared with CON group; #: P< 0.05; ##: P<0.01; ###: P<0.001; compared with HG group; n.s.: no significance.).

Based on animal experiments, we further investigated the effect and mechanism of Cana in protecting H9C2 cells against high glucose injury. Firstly, H9C2 cells were treated with different concentrations of Cana (0.5, 2.5, 5, 10, 20, 40 *μ*M) for 24 h with high glucose medium, and the cell viability was detected by CCK-8 kit. The result showed that, due to high glucose injury, the cell viability of H9C2 cells in HG group decreased by about 20% compared to normal cells. And compared with HG group, Cana could effectively improve the viability of H9C2 cells in a dose-dependent manner at a certain concentration range (0.5-10 *μ*M) (**Figure 3A**). Therefore, 10 *μ*M Cana with the best inhibitory effect on high glucose injury was selected for subsequent study.

Given the hypoglycemic effect of Cana in mice, the changes of intracellular glucose content with or without Cana were examined. Interestingly, our result found that Cana failed to effectively reduce the glucose level in H9C2 cells (**Figure 3B**), which may be related to tissue specificity as well as duration of action. In conclusion, Cana may have a non-hypoglycemic way to alleviate the high glucose injury of cardiomyocytes.

### Cana improves oxidative stress and mitochondrial damage induced by high glucose

**Figure 4:**
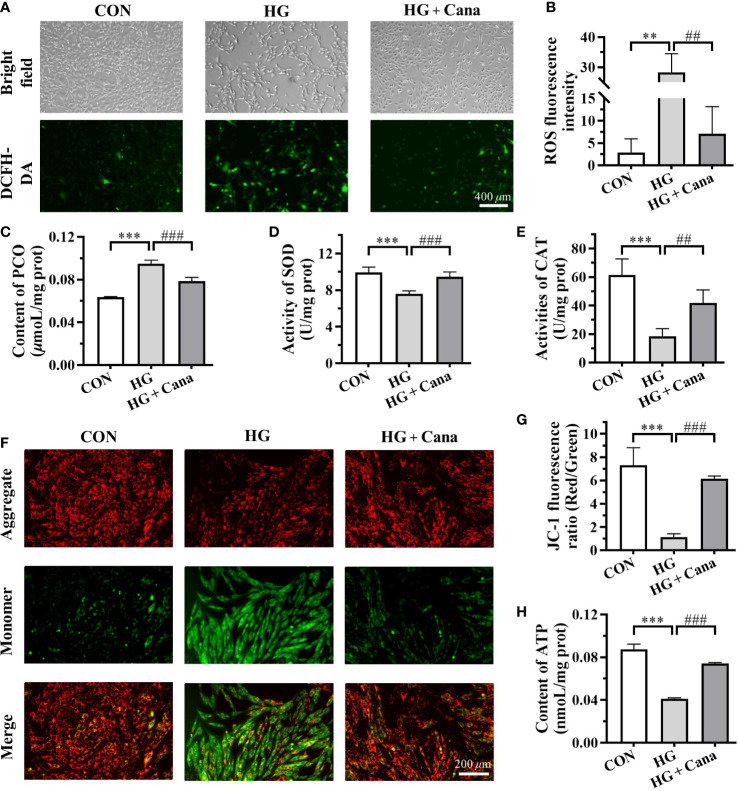
Canagliflozin improves oxidative stress and mitochondrial damage induced by high glucose. **(A)** The images and **(B)** fluorescence intensity of intracellular ROS stained by DCFH-DA (Scale bar: 400 *µ*m). **(C)** Content of PCO; Activities of **(D)** SOD and **(E)** CAT in H9C2 cells of each group. **(F)** The images and **(G)** JC-1 fluorescence ratio of MMP analyzed by JC-1 kit (Scale bar: 200 *µ*m). **(H)** Content of ATP. ROS, reactive oxygen species; PCO, protein carbonyl; SOD, superoxide dismutase; CAT, catalase; MMP, mitochondrial membrane potential; ATP, adenosine triphosphate. (**: P<0.01; ***: P<0.001; compared with CON group; ##: P<0.01; ###: P<0.001; compared with HG group.).

To determine the effect of Cana on oxidative stress in cardiomyocytes, the levels of ROS, PCO, SOD, and CAT in H9C2 cells were measured. As shown in **Figures 4A–E**, ROS Level and PCO content were significantly higher, whereas SOD and CAT activities were significantly declined in HG group cells than in CON. Like those in animal experiments, Cana could reverse the oxidative stress induced by high glucose by reducing intracellular ROS (**Figures 4A, B**) and PCO level (**Figure 4C**), and increasing intracellular antioxidant enzymes activities (**Figures 4D, E**).

As the main organelle of energy metabolism and ROS production, mitochondria play an important role in cardiomyocytes. In our study, it was found that high glucose intervention in H9C2 cells resulted in a decrease in MMP (**Figures 4F, G**) and ATP production (**Figure 4H**), leading to mitochondrial damage. Moreover, the high glucose-induced reduction in MMP (**Figures 4F, G**) and ATP production (**Figure 4H**) could be ameliorated by Cana. These results manifested that Cana had an antioxidant effect as well as a protective function of mitochondria.

### Cana mitigates ferroptosis induced by high glucose in cardiomyocytes

**Figure 5:**
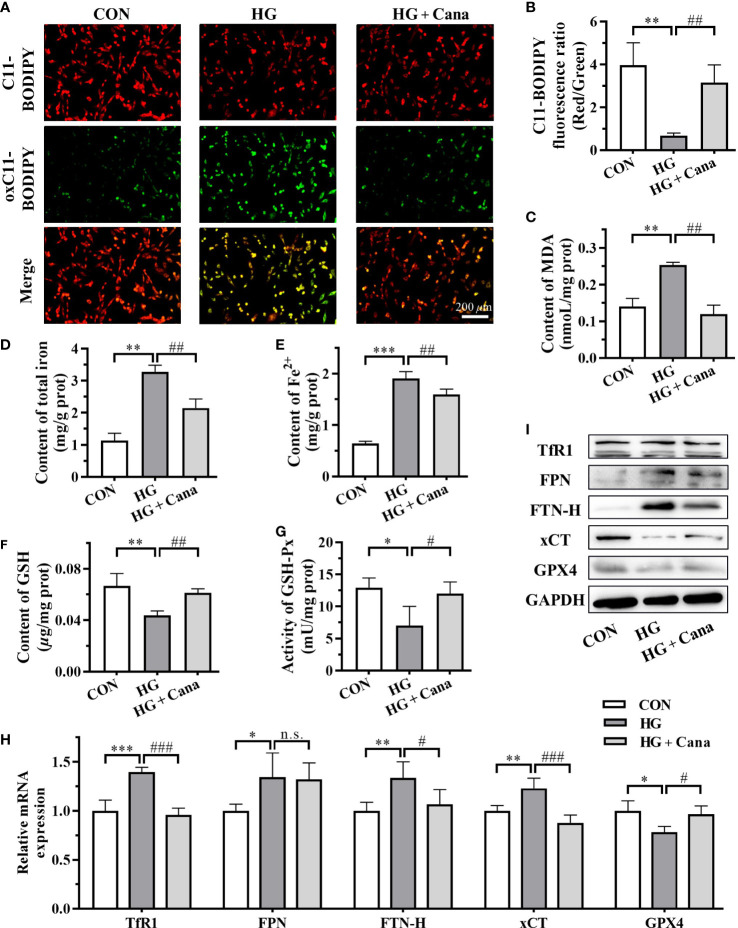
Cana mitigates ferroptosis induced by high glucose in cardiomyocytes. **(A)** The images and **(B)** C11-BODIPY fluorescence ratio of lipid ROS (Scale bar: 200 *µ*m). C11-BODIPY represents the level of staining with the probe (unoxidized), while oxC11- BODIPY (oxidized) represents the level of lipid ROS. Contents of **(C)** MDA, **(D)** total iron and **(E)** Fe^2+^ in H9C2 cells. **(F)** Content of GSH; **(G)** Activity of GSH-Px in H9C2 cells. **(H)** Transcriptional level and **(I)** protein level of ferroptosis related genes examined by qPCR and western blotting assay. ROS: reactive oxygen species; MDA: malondialdehyde. ROS, reactive oxygen species; MDA, malondialdehyde; GSH, glutathione; GSH-Px, glutathione peroxidase; FTN-H, ferritin heavy-chain; xCT, cystine-glutamate antiporter; GPX4, glutathione peroxidase 4; GAPDH, glyceraldehyde-3-phosphate dehydrogenase. (*: P<0.05; **: P<0.01; ***: P<0.001; compared with CON group; #: P<0.05; ##: P<0.01; ###: P<0.001; compared with HG group; n.s.: no significance.).

Since Cana maintained myocardial iron homeostasis in DCM mice, we further examined iron metabolism in H9C2 cells. After high glucose induction, iron deposition also observed in H9C2 cells, such as the increase of total iron (**Figure 5D**) and Fe^2+^ contents (**Figure 5E**) as well as FPN and FTN-H expression (**Figures 5H, I**). Although the mRNA expression of TfR1 was higher in HG group, the TfR1 protein level represented little difference in these three groups (**Figures 5H, I**). And after the intervention of Cana, the deposition of iron induced by high glucose was significantly reduced (**Figures 5D, E, H, I**).

The occurrence of ferroptosis is often accompanied by excessive accumulation of intracellular lipid peroxidation. C11-BODIPY staining showed that the level of oxC11-BODIPY was significantly increased in HG group compared with CON group (**Figures 5A, B**). At the same time, the increase of intracellular MDA level (**Figure 5C**) also reflected that high glucose induction increased the level of lipid ROS in cells of HG group. Furthermore, the system Xc^-^/GSH/GPX4 axis, a crucial pathway against lipid peroxidation and ferroptosis, was as well suppressed in HG group (**Figures 5F–I**). Among them, the high expression of xCT mRNA might be compensatory. After Cana intervention, the levels of GSH, GSH-Px and xCT expression inhibited by high glucose were effectively restored (**Figures 5F–I**), resulting in a decrease in lipid peroxidation and inhibition of ferroptosis. The results demonstrate that Cana ameliorated ferroptosis by regulating iron metabolism and system Xc^-^/GSH/GPX4 axis, which is consistent with the animal results.

## Discussion

Iron is an essential trace element in almost all living organisms, as it is involved in many important biological processes such as energy and metabolism, being critical for maintaining body homoeostasis. The evidence to date suggests that the maintenance of iron homeostasis is essential for proper cardiac function (7). When iron overload occurs in cardiomyocytes, the labile forms of iron enter the mitochondria and injure cells *via* oxidative damage, resulting in heart diseases (23). Further research confirmed that the pathogenic mechanisms of iron overload-induced cardiomyopathy is directly driven by ferroptosis (24), a new form of programmed cell death that was characterized by the overwhelming, iron-dependent accumulation of lethal lipid peroxidation (25). Additionally, an increasing number of evidence show that ferroptosis is also involved in the development of many cardiovascular diseases. As supported, ferroptosis inhibitors Vitamin E and coenzyme Q10 can protect the myocardium in diabetic animals by attenuating oxidative stress (26, 27), which indicated that ferroptosis is more likely to be involved in DCM. Recently, some direct evidences have been found to support the point that ferroptosis plays an essential role in the pathogenesis of DCM, but the exact mechanisms underlying this process are not clear. An interesting study demonstrated that T1DM-induced autophagy inhibition may activate Nrf2-mediated ferroptosis in cardiomyocytes, thereby contributing to the progression of DCM (9). Conversely, Cai group (8) identified that inhibition of ferroptosis prevented the development of myocardial dysfunction in the heart of T2DM mice and Nrf2 activation sulforaphane suppressed ferroptosis and prevented DCM by upregulating ferritin and xCT levels. Different from apoptosis, which mainly occurs at the early stage of DCM and decreases with the progression of DCM (28, 29), ferroptosis seems to be more important at the later stage of DCM (8). Therefore, targeting ferroptosis may serve as a feasible therapeutic approach for DCM prevention.

SGLT2is are a class of oral antidiabetic drugs that promote urinary glucose excretion by inhibiting renal proximal tubules from reabsorbing glucose, thus reducing blood glucose levels. Several large-scale randomized clinical trials have demonstrated that SGLT2is can improve cardiovascular outcomes in patients with or without DM (11, 30). These benefits have long moved beyond its role as antidiabetic drugs, which bring SGLT2is to the forefront. Recent studies have also found that SGLT2is can modulate iron metabolism and maintain body iron homeostasis as well as cardiac iron homeostasis (31, 32). Here, the role of ferroptosis in protecting myocardial function by SGLT2is has attracted more and more attention. As the first SGLT2i approved by FDA, Cana has been reported to be the most effective in preventing hospitalization for heart failure compared with other SGLT2is (33).

Several studies have manifested that Cana can regulate mitochondrial function *via* PPARα or AMPK-Sirt1-Pgc-1α signalling pathway (34). Meanwhile, as mentioned previously, Cana has several cardioprotective effects. Kondo et al (35) demonstrated for the first time that Cana suppressed myocardial NADPH oxidase activity and improved NOS coupling *via* SGLT1/AMPK/Rac1 signalling, leading to global anti-inflammatory and anti-apoptotic effects in the human myocardium. In experimental autoimmune myocarditis, Cana treatment markedly alleviated cardiac inflammation and improved cardiac function (13). And Cana also shown protective effects on diabetic hearts by inhibiting the mTOR/HIF-1α pathway to attenuate lipotoxicity in cardiomyocytes (20). Despite its cardioprotective effect is effective against isoprenaline-induced cardiomyocyte injury (14), but not for pirarubicin-induced cardiomyocyte injury (36). So further in-depth study is needed. Notably, a recent study reported that Cana exert its cardiovascular benefits partly *via* its mitigation of ferroptosis in a rodent model of HFpEF (15), which provide a new idea for the research of SGLT2is in heart disease, even DCM. So far, the cardioprotective mechanisms of Cana on DCM are not fully clear and needs to be further studied.

In our study, DM mice and H9C2 cells, a widely used cell line for the study of cardiac diseases (37), were used to establish DCM models *in vivo* and *in vitro*. Although Cana showed hypoglycemic effects in DCM mice, it did not appear to affect cardiomyocytes in the short term (24 h). In other studies, it was also mentioned that the cardioprotective effect of Cana may be independent of its hypoglycemic activity (38). Thus, there may be a non-hypoglycemic way of Cana to alleviate the high glucose injury of cardiomyocytes. Oxidative stress has been proved to play a significant role in the process of diabetes and its complications (39). Consistent with previous research (40, 41), hyperglycemia or high glucose did aggravate oxidative stress and induce cardiomyocytes injury in DM mice and H9C2 cells, indicating that the DCM model had been successfully established. In addition, the increase of total iron and Fe^2+^ contents and FTN-H expression reflected the occurrence of iron deposition in DCM models. *In vivo*, iron metabolism also regulated by hepcidin (31), which may account for the differences between animals and cells. At the same time, we noted a significant increase in MDA level with a remarkably decrease in GSH level in DCM models. As the substrate of GPX4, GSH not only play an antioxidant role but also participate in the occurrence of ferroptosis (42). The high level of MDA, one of the most important products of lipid peroxidation, reflected intracellular lipid ROS excessive accumulation. Besides, the fluorescence intensity of oxC11-BODIPY in H9C2 cells was dramatically increased after high glucose induction, which also proved that there were excessive lipid ROS in the cells. Hence, based on the fact that excess Fe^2+^ and lipid ROS exist, the main characteristics of ferroptosis, we hypothesized that ferroptosis does occur in the DCM models. In our study, Cana treatment mitigated ferroptosis by inhibiting myocardial oxidative stress and iron overload *in vitro* and *in vivo*. Moreover, in terms of morphological and structural changes, cells undergoing ferroptosis can be observed in TEM for mitochondrial abnormalities such as swelling, density changes and outer membrane rupture (25). These changes were indeed appeared in the cardiac tissue of DCM mice in our study, and Cana treatment ameliorated the damage of mitochondrial. All of these data indicated that ferroptosis may be one of the pathogeneses in DCM and Cana was able to palliate from ferroptosis to protect myocardial cells.

**Figure 6:**
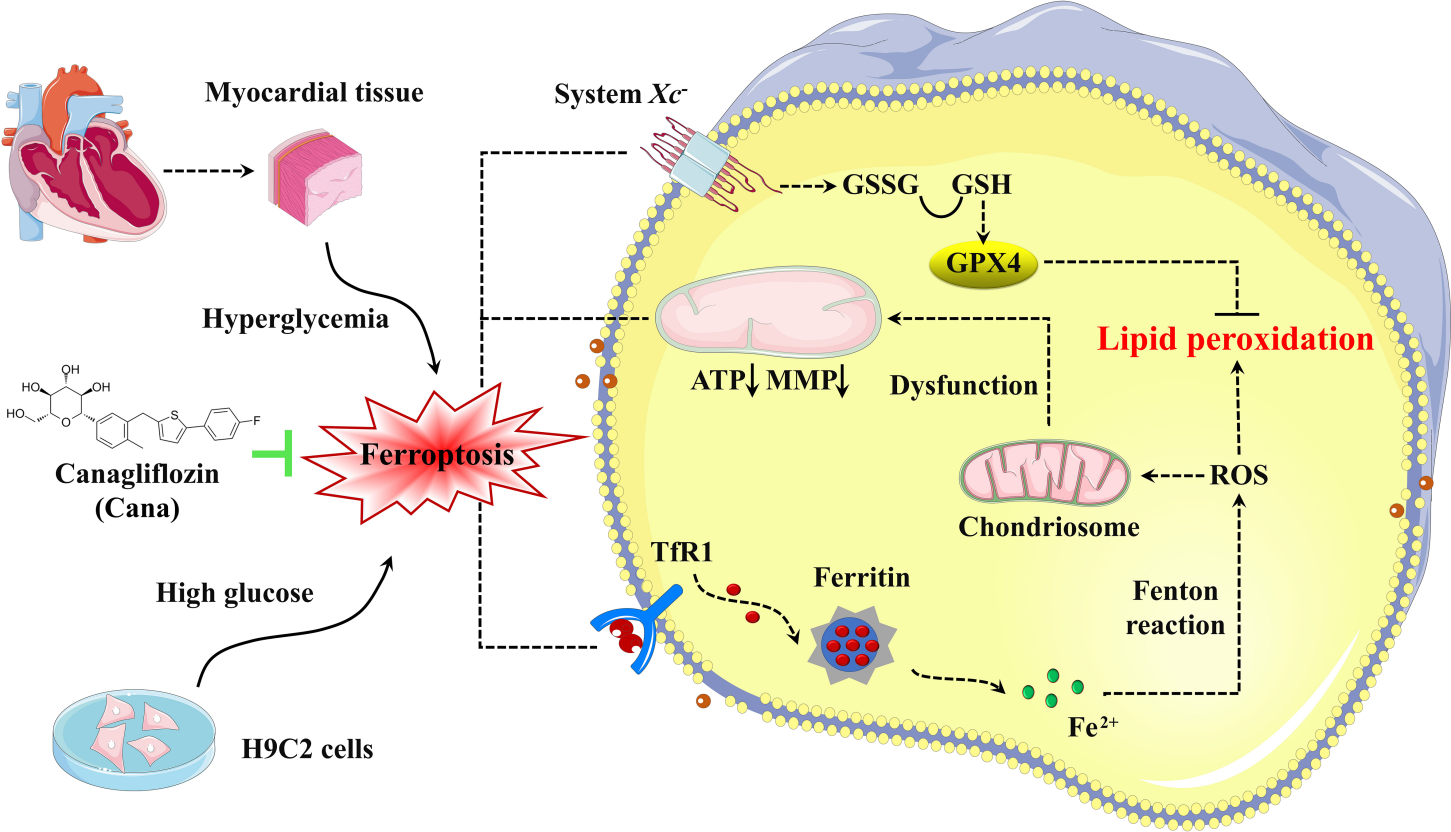
Schematic illustration of Cana alleviating ferroptosis in diabetic cardiomyopathy. High glucose, as pivotal pathogenic factors of DCM, inhibit the expression of SLC7A11 and promote the expression of TfR1, which in turn decrease the GSH levels and increase the labile iron levels, respectively. These alterations lead to increased lipid peroxidation and cardiomyocyte ferroptosis, which then initiates cardiac dysfunction, eventually leads to the DCM. Cana promotes upregulation of SLC7A11 and downregulation of TfR1 and FTN-H, which protect the cardiomyocytes from ferroptosis. TfR1, transferrin receptor 1; ROS, Reactive Oxygen Species; GSSG, oxidized glutathione; GSH, glutathione; GPX4, glutathione peroxidase 4; MMP, mitochondrial membrane potential; ATP, adenosine triphosphate.

At the cellular level, the initiation and execution of ferroptosis are controlled by the pathways involved in iron, amino acids and lipid metabolism. Briefly, iron, transported into cells by TfR1, is either stored in FTN-H or exported by FPN. The excess cellular iron, particularly Fe^2+^, can react directly with cellular oxidants to produce cytotoxic hydroxyl radicals *via* the Fenton reaction, which in turn promotes ferroptosis (7). The system Xc^-^/GSH/GPX4 axis is the main cellular pathway to protect cells from undergoing ferroptosis by catalyzing toxic lipid hydroperoxides into nontoxic lipid alcohols under normal physiological conditions (43). To initially explore the mechanism by which Cana alleviates ferroptosis, we performed an analysis of ferroptosis-related gene expression. Results showed when DCM occurred, system Xc^-^/GSH/GPX4 axis was suppressed, especially the xCT expression, while TfR1 and FTN-H were overexpressed. Similar results have also been found in myocardial tissue of mice with T2DM-induced DCM and rats with HFpEF (15). And after the treatment of Cana, the changes of these genes were partly reversed. Based on current evidences, we conclude that Cana may regulate ferroptosis to modify DCM by balancing cardiac iron homeostasis and overexpressing xCT (**Figure 6**). Further, it has been reported that Cana can activate AMPK/Nrf2/ATF4 pathway and inhibit p53 expression (44, 45). Nuclear factor erythroid 2-related factor 2 (Nrf2) and activating transcription factor 4 (ATF4) activated xCT expression by interacting with its promoter, while the deubiquitination of histone H2B by p53 inhibited xCT expression (46). For TfR1, Cana has been reported to inhibit mechanistic target of rapamycin (mTOR) (20), and the inhibition of mTOR could mediate the degradation of TfR1 mRNA through tristetraprolin (TTP). However, the relationship and specific mechanism of Cana in regulating iron metabolism and system Xc^-^/GSH/GPX4 axis still need to be studied in depth, which will be the focus of our next attention.

The efficacy of SGLT2is as an adjuvant for insulin therapy in patients with T1DM has been verified in several clinical trials (47, 48). Despite the potential benefits in people with T1DM, current researches on Cana mainly focus on T2DM. And rare relevant studies have identified the role of Cana in the treatment of T1DM induced cardiomyopathy. Our animal experiment found that Cana can really control blood glucose and diabetic symptoms in T1DM mice, and even attenuated myocardial injury through non-hypoglycemic way. Hence, Cana is a promising agent as an adjuvant for insulin therapy in the prevention and treatment of DCM. However, its potential mechanism and clinical safety need further exploration.

## Data availability statement

The original contributions presented in the study are included in the article/supplementary material. Further inquiries can be directed to the corresponding author.

## Ethics statement

Experiments were carried out according to the Guideline for the Care and Use of Laboratory Animals published by the National Institute of Health, USA. All experimental procedures were reviewed and approved by the Animal Experimental Ethics Committee of Zhejiang University of Technology (Animal experimental research plan No. 20220317021).

## Author contributions

SD, HS, LX, PW and YS conceived and designed this research. SD performed experiments, analyzed data, and drafted the manuscript. All authors interpreted results of experiments. HS, LX, YS provided help on the edition of figures. HS, YS and LX edited and revised the manuscript. All authors contributed to the discussion, read and approved the final manuscript. All authors contributed to the article and approved the submitted version.

## Funding

This work was supported by grants from Medicine and Health Science and Technology Plan Projects of Zhejiang Province (YS, 2020PY029), Science and Technology Innovation Special Project of Jiaxing Science and Technology Bureau (YS, 2020AY30003), Zhejiang Provincial Health Science and Technology Program of Traditional Chinese Medicine (YS, 2021ZB283) and Jiaxing Key Laboratory of Diabetic Angiopathy.

## Acknowledgments

The authors thank Prof. Zhu-FY, Prof. Wang-B and Prof. Tai-Y (Electron Microscope Platform, Medical Research Center of Zhejiang Chinese Medicine University, Hangzhou, China) for providing the technical support of TEM; Prof. Chen-Y, Prof. Wang-SF (Experimental Animal Center of Zhejiang University of Technology, Hangzhou, China) for providing the technical support of electrocardiogram.

## Conflict of interest

The authors declare that the research was conducted in the absence of any commercial or financial relationships that could be construed as a potential conflict of interest.

## Publisher’s note

All claims expressed in this article are solely those of the authors and do not necessarily represent those of their affiliated organizations, or those of the publisher, the editors and the reviewers. Any product that may be evaluated in this article, or claim that may be made by its manufacturer, is not guaranteed or endorsed by the publisher.
